# The effectiveness of adding cognitive behavioural therapy aimed at changing lifestyle to managed diabetes care for patients with type 2 diabetes: design of a randomised controlled trial

**DOI:** 10.1186/1471-2458-7-74

**Published:** 2007-05-08

**Authors:** Laura MC Welschen, Patricia van Oppen, Jacqueline M Dekker, Lex M Bouter, Wim AB Stalman, Giel Nijpels

**Affiliations:** 1 EMGO Institute, VU University Medical Center, Amsterdam, the Netherlands; 2 Department of General Practice, VU University Medical Center, Amsterdam, the Netherlands; 3 Department of Psychiatry, VU University Medical Center, Amsterdam, The Netherlands

## Abstract

**Background:**

In patients with type 2 diabetes, the risk for cardiovascular disease is substantial. To achieve a more favourable risk profile, lifestyle changes on diet, physical activity and smoking status are needed. This will involve changes in behaviour, which is difficult to achieve. Cognitive behavioural therapies focussing on self-management have been shown to be effective. We have developed an intervention combining techniques of Motivational Interviewing (MI) and Problem Solving Treatment (PST). The aim of our study is to investigate if adding a combined behavioural intervention to managed care, is effective in achieving changes in lifestyle and cardiovascular risk profile.

**Methods:**

Patients with type 2 diabetes will be selected from general practices (n = 13), who are participating in a managed diabetes care system. Patients will be randomised into an intervention group receiving cognitive behaviour therapy (CBT) in addition to managed care, and a control group that will receive managed care only. The CBT consists of three to six individual sessions of 30 minutes to increase the patient's motivation, by using principles of MI, and ability to change their lifestyle, by using PST. The first session will start with a risk assessment of diabetes complications that will be used to focus the intervention.

The primary outcome measure is the difference between intervention and control group in change in cardiovascular risk score. For this purpose blood pressure, HbA_1c_, total and HDL-cholesterol and smoking status will be assessed. Secondary outcome measures are quality of life, patient satisfaction, physical activity, eating behaviour, smoking status, depression and determinants of behaviour change. Differences between changes in the two groups will be analysed according to the intention-to-treat principle, with 95% confidence intervals. The power calculation is based on the risk for cardiovascular disease and we calculated that 97 patients should be included in every group.

**Discussion:**

Cognitive behavioural therapy may improve self-management and thus strengthen managed diabetes care. This should result in changes in lifestyle and cardiovascular risk profile. In addition, we also expect an improvement of quality of life and patient satisfaction.

**Trial registration:**

Current Controlled Trials ISRCTN12666286

## Background

Diabetes mellitus is a major health problem. It was estimated that in 2000 approximately 177 million people worldwide had diabetes, and this number is expected to double by the year 2030 [1]. Cardiovascular disease is the leading cause of death among patients with diabetes. Both cardiovascular disease and diabetes are associated with similar risk factors, namely unhealthy diet, smoking and a sedentary lifestyle. These factors contribute to a severely elevated morbidity and mortality of patients with diabetes [2-5]. In the last few years it has been shown that interventions can lower these risk factors and improve cardiovascular and microvascular endpoints [6-10].

In previous studies it has been shown that different behavioural interventions are effective in changing lifestyle of patients with diabetes [11-14]. However, it is not entirely clear what kind of therapy is most effective [15]. We believe that a combined behavioural intervention, consisting of a motivational phase and problem solving techniques that is tailored to lifestyle determinants has to be used [11-14]. To our knowledge, this has not yet been studied.

The aim of our study is to evaluate if adding a combined behavioural intervention to managed diabetes care, is effective in improving the cardiovascular risk profile of patients with type 2 diabetes.

### Diabetes Care System (DCS)

In addition to the care of the GPs and their practice nurses, in West-Friesland, the Netherlands, an extensive diabetes management programme, is implemented [16]. In short, each patient visits annually the diabetes research center for a physical examination. In addition, the patient visits a diabetes nurse and a dietician for information and advice and is invited for follow-up visits when necessary. The results of this annual examination are sent to the patient's GP, who is responsible for the management of the patient and delegation of tasks to their practice nurse, according to the guidelines of the Dutch College of General Practitioners. These guidelines recommend every patient with diabetes to visit a GP every 3 months [17,18].

At present, 3800 patients, representing 80% of all type 2 diabetes patients in the sub-region West-Friesland are enrolled in this Diabetes Care System (DCS). The mean HbA_1c _of these patients was 7.0% in 2005. However, according to current guidelines of the Dutch College of General Practitioners [18] more than 60% of the patients participating in the DCS still had a HbA_1c _≥ 7.0% and 90% of the patients had a HbA_1c _≥ 8.5%. In addition, 20% of the patients still smoked, 80% was overweight and 70% had little or no physical activity. These findings show that the DCS was successful in controlling hyperglycaemia, but failed with respect to the lifestyle factors. Our findings are similar to those of other diabetes management programs [19]. This implies that there is a need for innovative programmes to improve the quality of care for diabetes patients, with a specific focus on lifestyle factors.

### Cognitive behavioural therapy (CBT)

During the last few decades, several models have been developed to explain and predict health behaviour. A common used model is the Atittude, Social influence, and self-Efficacy model (ASE-model) [20-22]. It mainly incorporates the attitudes and social influences determinants of the Theory of Reasoned Action and the self-efficacy concept of Bandura's Social Cognitive Theory [23]. This model has proven to be effective for developing behavioural interventions in different research areas and will be used in our study to evaluate if the determinants of behaviour of the patient have changed after the CBT [24-26].

The ASE-model assumes that behaviour has three primary determinants: attitudes, social influences and self-efficacy. Together, they determine the intention of a person to perform a specific behaviour. In this model, a person's attitude towards a specific behaviour is determined by its perceived affective (evaluative) and cognitive consequences. The social influences are determined by social norms, perceptions of the behaviour of others and perceived social support or pressure for execution of the behaviour. The self-efficacy expectations can be seen as a person's belief in his or hers ability to perform the desired behaviour in specific social, emotional and habitual situations. These three determinants are not entirely independent of each other; they can all be seen as cognitions that the person has regarding the importance of execution of a specific behaviour.

We developed a CBT that consists of two parts: Motivational Interviewing and Problem Solving Treatment.

#### Motivational Interviewing (MI)

MI is a client-oriented, directive method for enhancing intrinsic motivation to change by exploring and resolving ambivalence [27]. The four guiding principles of MI are: express empathy, develop discrepancies, roll with resistance and support self-efficacy.

Expressing empathy involves providing clients with an atmosphere of respect and acceptance of their position. The technique used is reflective listening and this is generally considered the foundation of MI and is recommended throughout the whole counselling process.

The second principle of MI involves creating a 'gap' between the client's current behaviour and their broader goals, thus cultivating motivation for lifestyle change. When the client recognizes such discrepancies, a certain level of discontent arises that makes change more likely to occur. Discrepancies are developed by exploring the client's important life values and reviewing how their current behaviours affect their ideal lifestyle.

The third principle, rolling with resistance, is based on the idea that directly challenging resistance is counterproductive to lifestyle change, because it typically results in the client defending the current state of affairs. Rather, resistance should be rolled with and channelled instead of confronted. Rolling with resistance invites the client to consider a new perspective versus having it imposed.

Finally, self-efficacy, or one's confidence in the ability to change a specific behaviour under difficult circumstances, should be supported whenever possible because it is one of the best predictors of treatment outcome. Self-efficacy can be strengthened by affirming past success (i.e., reinforcement), presenting success stories of others (i.e., modelling), and expressing belief in the client's potential to change.

Together, these four principles guide the first phase of CBT. The patient will become motivated and at that point, the Problem Solving Treatment can be started.

#### Problem Solving Treatment (PST)

PST is a practical skill building treatment and might therefore be effective in achieving lifestyle changes in patients with diabetes [28].

PST was originally described by D'Zurilla and Godfried [29] and later expanded and refined by D'Zurilla and Nezu [30]. PST may be defined as the self-directed cognitive-behavioural process by which a person attempts to identify effective or adaptive solutions for specific problems encountered in everyday living [31]. The effects of PST have been shown to be effective in patients with depression [32-34]. However, PST is potentially useful in the treatment of any disorder in which a patient is experiencing multiple daily problems and stresses and is not coping effectively with them [31]. Therefore, patients with diabetes might benefit from PST as they have to cope with many complex tasks. They have to take care of their diet, lifestyle, physical activity and administration of oral medication or insulin in order to achieve an optimal glycaemic control and to reduce their cardiovascular risk factors. Also, psychological problems are common in these patients. PST might increase the patients' ability to solve their problems in a structured way and their confidence in facing future problems and therefore improve their diabetes self-management.

PST can be considered as a series of stages [28]:

1. Explanation of the intervention and its rationale

2. Definition and breaking down of the problem

3. Establishing achievable goals for problem resolution. Achievable goals are SMART goals: Specific, Measurable, Achievable, Relevant, Timed

4. Generating multiple possible solutions

5. Evaluating and choosing the solution

6. Implementing the preferred solution

7. Evaluating the outcome

Another reason why PST may be suitable in patients with diabetes is that practice nurses can deliver it because it is relatively brief, as it lasts between three and six sessions of 30 minutes [32,35].

### Objectives

The primary objective of our study is to investigate if cognitive behavioural therapy, when added to managed diabetes care, is effective in achieving changes in cardiovascular risk profile. The secondary objective is to assess whether the cognitive behavioural therapy is effective in changing lifestyle.

## Methods

### Design of the study

The study is a randomised controlled trial (RCT). The Medical Ethical Committee of the VU University Medical Center in Amsterdam approved the study design, protocols, information letters to the patients and informed consent form.

### Setting

The trial is conducted within 'the Diabetes Care System (DCS)', a managed care system that was implemented in The Netherlands, as described above. This care system has a well organised infrastructure with experienced medical assistants, diabetes nurses and dieticians and is therefore a suitable setting to implement a new therapy for patients with diabetes.

### Study population

The source population consist of patients with type 2 diabetes of participating GPs. Selection criteria for the GPs are that they have a practice nurse and that they are part of the DCS. GPs will be informed about the study by one of the researchers (GN) and a diabetes nurse and are asked to participate. They have to sign an intention declaration to confirm participation.

Patients from the participating GPs, who are scheduled to visit the DCS for their annual visit, are sent an information letter on the study. This letter is announced in the telephone call that the reception of the DCS has with the patient to make an appointment for their annual visit at the medical assistant.

Patients visit the medical assistant for their annual physical examination of the DCS. The medical assistant checks eligibility of the patient for the study. Patients are eligible if they meet the following inclusion criteria: diagnosis of type 2 diabetes, between 40 and 75 years of age, able to understand the Dutch language in order to understand the study protocol, written informed consent and questionnaires, high risk for cardiovascular disease and diabetic complications. Patients are considered at risk if, according to measurements of the previous annual visit, HbA_1c _≥ 7.0 % and/or body mass index ≥ 27.0 kg/m^2 ^and/or the patient smokes. In addition, all newly diagnosed patients, who do not have annual check-up results available, are considered eligible, based on the fact that all new patients need to learn how to deal with their diabetes. If all inclusion criteria are met, the medical assistant explains the purpose of the study and patients are asked if they are interested in participation.

All patients must sign a written informed consent form. Subsequently, physical measurements are performed, which include body weight, waist circumference, blood pressure, and drawing of blood samples (to assess fasting blood glucose, HbA1c, total cholesterol, HDL-cholesterol and triglycerides). In addition, patients are given a self-administrated questionnaire to fill in at home, including questionnaires on physical activity, dietary intake, smoking status, determinants of behaviour according to the ASE-model, quality of life, depression, general health and patient satisfaction. The responsible GP is informed that the patient is participating in the study. During the study, GPs receive fax forms from the diabetes nurses and dieticians advising on required medication changes for a specific patient.

### Treatment allocation

Patients are randomly assigned to either the intervention group, receiving CBT in addition to the managed diabetes care or the control group, receiving managed diabetes care only. Approximately three weeks after the visit to the medical assistant, patients are scheduled for either the first intervention or control session to the diabetes nurse and dietician.

**Figure 1:**
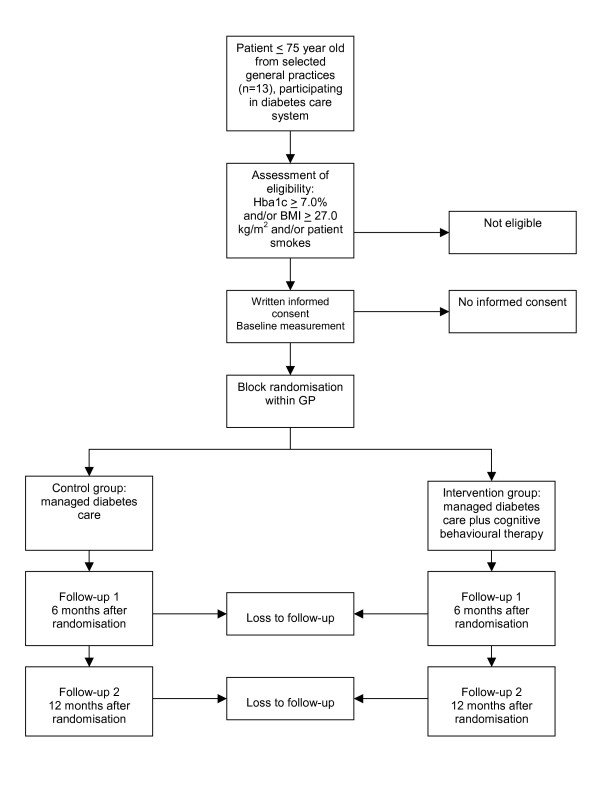
Design of the RCT.

Block randomisation is performed within the general practices in order to eliminate the influence of different treatments of general practitioners. From each general practice, half of the patients are randomised to the intervention group or the control group. The principle investigator (LMCW), who is not involved in the selection and treatment of patients, prepared a randomisation list using series of random numbers [36]. Random permuted blocks of 4 patients are made to ensure equal distribution of patients for each general practice. The manager of the DCS, who is not involved in the patients' care, allocates the patient to one of the two groups by using the randomisation list. The flow of the patients is registered by the principal investigator (LMCW), according to a flow diagram recommended by the CONSORT statement [37]. Reasons for withdrawal are registered by the medical assistant. Figure 1 shows the design of the study.

### Blinding

Patients, diabetes nurses and dieticians cannot be blinded to the intervention. The medical assistants are blinded as far as possible. They have close contact with the diabetes nurses and dieticians within the DCS and therefore we cannot guarantee the blinding of the medical assistants. However, they are not involved in the intervention.

The principal investigator, the GPs and their practices nurses remain blinded during the entire intervention. This is achieved by giving instructions to the patients preferably not to communicate about the intervention with their GPs and practice nurses. To evaluate the success of blinding, GPs will be asked to fill in a questionnaire to investigate to which group each of their participating patients they believed were allocated.

### Interventions

#### Control group

Patients allocated to the control group receive managed diabetes care provided by the DCS.

This managed diabetes care consists of three elements. Firstly, these patients are invited by means of a telephone call from the reception of the DCS to make an appointment for an annual physical examination at the medical assistant. Secondly, the patient visits a diabetes nurse and dietician, for 30 minutes each, in order to receive general information and advice on diabetes problems, dietary intake and physical activity. The results of the physical examination and the recommendations of the diabetes nurses and dietician are sent to the patients' GP, in order to stimulate the GP to follow the guidelines of the Dutch College of General Practitioners [17,18]. Thirdly, the DCS coordinates the care of the patient between the general practices and secondary diabetes care.

Patients in the control group are scheduled for one visit to the diabetes nurse and for one visit to the dietician; follow-up visits are optional.

#### Intervention group

Patients who are assigned to the intervention group receive CBT in addition to the same managed care. The CBT is performed by diabetes nurses and dieticians, who received a training of two days in the performing of CBT prior to the onset of the study. Additionally, two instruction days on the implementation of the intervention were arranged. A treatment manual is used during the study to guide the treatment. All CBT sessions are tape-recorded to assess treatment fidelity of the diabetes nurse or dietician to the protocol. In addition, these tape-recordings are used in supervision sessions to provide ongoing feedback. Each diabetes nurse and dietician haves supervision once every four weeks with a psychologist (PvO) who is specialised in CBT. This psychologist is also available by email or telephone between the supervision sessions.

A pilot study was performed before the start of the study to provide three practice sessions for each diabetic nurse and dietician and to assess logistic pathways of the study. There were 10 caregivers involved in the study, which means that 30 patients were included in the pilot study. A supervision meeting was scheduled after the pilot study to discuss logistic pathways and skills to perform the CBT of every caregiver.

The CBT starts with a visit to the diabetes nurse. Results of the baseline measurements can be used to motivate the patient to change. With the help of MI techniques, a problem list is created by the patient focused on lifestyle (smoking behaviour, diet and physical activity). When the most important problem is related to smoking or physical activity, the patient visits the diabetes nurse during the next sessions. When the most important problem is related to diet, the patient is rescheduled to visit the dietician during follow-up sessions. From that moment, the patient remains with the same diabetes nurse or dietician during the entire study.

After the MI phase, is it time to strengthen commitment to a lifestyle change plan. At this point, the patient is willing and able to change. Signs of this readiness for change are: decreased resistance, decreased discussion about the problem, reaching some kind of resolution, increasing change talks, questions about the change, and envisioning how life might be after a change. At this time, the diabetes nurse or dietician makes, together with the patient, an implementation plan where, when and how the behaviour changes will be performed. The formulation of this plan makes it easier for patients to carry out intended actions. This will be done by PST [28-31].

The CBT consists of three to six visits of 30 minutes to the diabetes nurses or dieticians at the DCS. There is a focus on one problem area in each session. During all sessions, both the MI and the PST skills are used to motivate the patients, set new goals and review the patients' progress. The last CBT session is brought to an end by reviewing and emphasising the entire CBT process, with emphasis on achieved successes of the patient and implementation of CBT in the future, independently of the diabetes nurse or dietician. The GPs provide ongoing feedback during the intervention.

### Co-interventions and compliance

Co-interventions are avoided during the intervention by means of the fact that the control group was scheduled to a diabetes nurse that did not receive the training on CBT. In addition, all caregivers in the Diabetes Care System have access to the same central database which includes clinical characteristics of the patients and details on given advice and information. This centralisation avoids co-interventions. However, any co-intervention is reported. Compliance to the CBT is assessed by registration of the number of sessions that patients attend.

Compliance to the intervention is considered adequate when at least three sessions are attended. For the patients in the control group, the number of follow-up visits to the diabetes nurse or dietician is also registered. The number of visits to the GPs or hospital is registered for both the intervention and the control group.

### Outcome assessment

Outcome measurements are assessed at baseline, and again after 6 and 12 months by means of self-reported questionnaires and physical examinations at the DCS. Physical examinations are performed by medical assistants of the DCS. These included weight, height, waist circumference, blood pressure, fasting blood glucose, HbA1c, triglycerides (at baseline and 12 months only), total cholesterol (at baseline and 12 months only) and HDL-cholesterol (at baseline and 12 months only). Demographic variables on age, gender, diabetes duration, marital status, ethnicity, level of education and eventual occupation are assessed at baseline by using a self-administered questionnaire, which also includes all secondary outcomes. The medical assistants ask the patients information on medication (the patient brings the packages of medication to the visit), hospital admission days and number of general practitioner visits. Patients are informed of their measurements by the diabetes nurse and dietician. Measurements are also send to their GPs.

#### Primary outcome measures

The primary outcome measure is the difference between intervention and control group in change in cardiovascular risk score after 12 months. The cardiovascular risk score is based on the Oxford Risk Engine (algorithm that includes: age at diagnosis, duration of diabetes, sex, ethnicity, smoking status, systolic blood pressure, HbA_1c_, total cholesterol, HDL-cholesterol) [38].

#### Secondary outcomes measures

Secondary outcomes measures are included in a self-reported questionnaire that is filled in by the patients at home. Patients will be sent a questionnaire at baseline, after 6 and 12 months. All the secondary outcomes are included in the questionnaire, with the exception of the Patients' evaluation of the Quality of Diabetes Care Questionnaire (PEQD), which is not assessed at baseline.

1. Diet is assessed by the use of the Dutch Eating Behaviour Questionnaire (DEBQ). This is a 33-item questionnaire regarding diet behaviour, scored on a five point scale (1 = never, 2 = seldom, 3 = sometimes, 4 = often, 5 = very often). This questionnaire assess whether a patient is a restraint, emotional or external eater [39].

2. Short Questionnaire to Assess Health Enhancing Physical Activity (SQUASH) is used to assess physical activity. This questionnaire consists of subscales concerning (A) commuting activities, (B) leisure time activities, (C) household activities, and (D) activities at work and school [40].

3. Smoking: Smoking is assessed by asking patients if they are a never smoker, current smoker or past smoker.

4. ASE-questionnaire: a 20-item questionnaire, created by the investigators themselves according to the ASE-model: Attitude, Social influences and self-Efficacy model [41]. To our knowledge, no validated questionnaires were available. The theoretical model and questionnaires of other running studies at their own research institute were used to develop the ASE-questionnaire. Each item is provided with a 7-point Likert-type scale describing only the two end points.

5. Quality of life is measured by the EuroQol. This questionnaire consists of 5 dimensions (mobility, self-care, usual activities, pain/discomfort, and anxiety/depression), each with 3 levels, and a visual analogue scale on which patients rate their own health between 0 and 100 [42,43].

6. Depression during the last week is assessed by the use of the CES-D: Center for Epidemiological Studies Depression scale. This is a 20-item questionnaire with a four-point scale (1 = seldom or never, 2 = sometimes, 3 = often, 4 = mostly or always) [44].

7. The Patient Health Questionnaire (PHQ9) is used to assess the general health of the patient. This brief PHQ consists of 9 items, measured on a four-point scale (1 = not at all, 2 = several days, 3 = more than half of the days, 4 = almost every day) in order to assess depressive disorders during the last two weeks [45].

8. The patient satisfaction on the diabetes care provided by medical assistants, diabetes nurses and dieticians is measured by the use of the Patients' evaluation of the Quality of Diabetes Care Questionnaire (PEQD). For each health care provider, 14 items are evaluated on a five-point scale, ranging from "poor" to "excellent" [46].

#### Physical measurements

Physical measurements are performed to calculate the cardiovascular risk score and to describe the patients' characteristics.

1. Anthropometric measurements. Body weight and height are measured for the calculation of the Body Mass Index (weight divided by height squared). Waist circumference is measured at the level midway between the lowest rib margin and the iliac crest.

2. Systolic and diastolic blood pressure is measured after 5 minutes of rest in seated position by Collin Press Mate (BP-8800, Komaki-City).

3. Blood samples are taken in order to determine:

• fasting plasma glucose: measured in plasma by means of a hexokinase-method (Roche Diagnostics GmbH, Mannheim, Germany).

• HbA_1c_: measured by High Performance Liquid Chromatography

• Total cholesterol, HDL-cholesterol and triglycerides by means of enzymatic techniques (Boehringer-Mannheim, Mannheim, Germany).

4. In addition, data are collected from the patients on:

• hospital admission days per 6 months

• number of visits to general practitioner per 6 months

• medication changes per 6 months

### Sample size

The sample size is calculated on the base of the minimally clinically relevant difference in changes of one of the primary outcome measurements, the Oxford Risk Engine [38]. In the DCS the standard deviation of the absolute risk for cardiovascular disease, calculated with the Oxford Risk Engine, was 10%. A difference (d) between the intervention and control group in changes of 5% absolute risk (about the same as can be expected by giving a cholesterol lowering agent, i.e. a statin as medication), a standard deviation (sd) of 10%, an alpha of 0.05 (two-sided), and a power of 90% leads to 68 participants in each group. A multilevel analysis was performed to calculate the correlation of the outcome measures within practices. A correlation (r) of 20% was found and for that reason the number of participants has to be multiplied with a factor 0.8 (1 – correlation, to adjust for the block randomisation within general practices), leading to 68 patients (n) in each group. The formula used was [47,48]:

n1 = n2 = 2(Z_1-α/2 _+ Z_1-β_)^2 ^sd^2 ^(1-r)/d^2^

Z_1-α/2 _= Z (1 - 0.05/2) = Z 0.975 = 1.96

Z_1-β _= Z 0.90 = 1.282

sd = 0.10, r = 0.20, d = 0.05

n1 = n2 = 2 * (1.96 + 1.282)^2 ^* (sd*sd)* (1-r)/(d*d) =

2 * 10.51 * (0.1 * 0.1) * (1 - 0.2)/(0.05 * 0.05) = 68 patients. Taking into account a possible drop-out rate of 30% the number of participants to be randomised must be 194 (97 in each group).

### Analyses

Comparability between the two groups will be assessed at baseline. On the basis of an intention-to-treat analysis, differences in changes between the intervention and the control group are measured with 95% confidence intervals at 12 months for both primary and secondary outcomes. For dichotomous outcome variables a multilevel logistic regression analysis will be used. To calculate differences between continuous variables a multilevel analysis will be used, to adjust for the clustering of observations of patients receiving care from the same GP, and for repeated measurements within one patient. We will also perform a per-protocol analysis with patients that have attended at least three CBT sessions.

We will adjust data for possible confounders: age, gender, weight at start of study, diabetes duration, level of education, depression, smoking, number of attended sessions, general practitioner, and type of health care provider (diabetes nurse or dietician).

## Discussion

This article provides a detailed description of the study design of an RCT on the added value of cognitive behavioural therapy focused on changing lifestyle in patients with type 2 diabetes and is published with two purposes. Firstly, to provide the opportunity for other researchers, health care providers and policy makers to critically review the methodological quality, the background theory and the practical issues of the RCT. Secondly, the publication of this study design prevents publication bias. Studies with non-significant results are less likely to get published than studies with significant results. For that reason, the result of meta-analyses will be biased. The publication of our study design before the results become available will therefore prevent publication bias. The study becomes identifiable in search strategies for a systematic review and/or meta-analysis [49]. In addition, the authors bear more responsibility to publish the results, irrespective of the results appear to be positive or negative.

We expect that the addition of a cognitive behavioural therapy to managed diabetes care will be effective in changing lifestyle in patients with type 2 diabetes. As a result of that, we expect that a changed lifestyle will cause a risk reduction in cardiovascular disease. The implementation of such a study into an existing managed diabetes care system is unique. This system provides experienced medical assistants, diabetes nurses and dieticians and a high-quality infrastructure for patients with diabetes. One could argue, that, because the setting is well organised, the results of this trial cannot be easily generalised to all patients with diabetes. However, when the results appear to be negative, it is very unlikely that such an intervention will have success in a general setting.

There are some limitations in the study design. We expect that patients who agree to participate are more motivated to change than patients who refuse to participate. However, because the design of the study is a randomised controlled trial, this will be the case in both groups. Having a population of motivated patients might decrease the drop-out percentage. Patients in the control group may improve their lifestyle behaviour just by the knowledge that their outcomes are being used for research. However, this may also occur in the intervention group. Patients in the intervention group may benefit from the attention they get. However, because it is possible that patients in the control group receive follow-up visits as well, we can identify sub-groups based on number of sessions and compare the outcomes of the intervention and the control sub-groups in order to make a distinction between the effect of attention and the intervention.

Another limitation of this study is the lack of a validated questionnaire for an important primary outcome measure, the ASE-questionnaire. This questionnaire is separated into three dimensions (diet, physical activity and smoking). Because the use of validated questionnaires to assess the amount of physical activity, the diet behaviour and the amount of smoking, we can compare these outcomes with the ASE-questionnaire. We expected that these correlate with each other. It might therefore be possible to validate the ASE-questionnaire in this study. This might not only increase the quality of this study but can also provide a questionnaire for other researchers.

The study already started at the end of 2005. The inclusion of patients will take one year, till the end of 2006. The one-year follow-up period of all patients is finished at the end of 2007 and results will then become available. If this study appears to have positive effects, the behavioural intervention might also be implemented in other care settings.

## List of abbreviations used

ASE-model = Attitide, Social influences and self-Efficacy model

DCS = Diabetes Care System, West-Friesland, The Netherlands

CBT = Cognitive Behavioural Therapy

GP = General Practitioner

MI = Motivational Interviewing

PST = Problem Solving Treatment

## Competing interests

The authors declare that they have no competing interests.

## Authors' contributions

LMCW is responsible for the data collection and wrote the manuscript. GN, JMD and PvO developed the original idea for the study. The study design was further developed by GN, PvO, JMD and LMCW. All authors read and corrected draft versions, and approved final versions of the study protocol for the Medical Ethical Committee of the VU University Medical Center and of this manuscript.

## Pre-publication history

The pre-publication history for this paper can be accessed here:


